# A Fully Automated High-Throughput Training System for Rodents

**DOI:** 10.1371/journal.pone.0083171

**Published:** 2013-12-06

**Authors:** Rajesh Poddar, Risa Kawai, Bence P. Ölveczky

**Affiliations:** 1 Department of Organismic and Evolutionary Biology, Harvard University, Cambridge, Massachusetts, United States of America; 2 Center for Brain Science, Harvard University, Cambridge, Massachusetts, United States of America; MRC-National Institute for Medical Research, United Kingdom

## Abstract

Addressing the neural mechanisms underlying complex learned behaviors requires training animals in well-controlled tasks, an often time-consuming and labor-intensive process that can severely limit the feasibility of such studies. To overcome this constraint, we developed a fully computer-controlled general purpose system for high-throughput training of rodents. By standardizing and automating the implementation of predefined training protocols within the animal’s home-cage our system dramatically reduces the efforts involved in animal training while also removing human errors and biases from the process. We deployed this system to train rats in a variety of sensorimotor tasks, achieving learning rates comparable to existing, but more laborious, methods. By incrementally and systematically increasing the difficulty of the task over weeks of training, rats were able to master motor tasks that, in complexity and structure, resemble ones used in primate studies of motor sequence learning. By enabling fully automated training of rodents in a home-cage setting this low-cost and modular system increases the utility of rodents for studying the neural underpinnings of a variety of complex behaviors.

## Introduction

Studies exploring the neural mechanisms underlying higher-order cognitive and learning phenomena, including decision making[1], motor skill execution[2], and perceptual discrimination[3,4], have traditionally been done in non-human primates. Costs and regulations, however, make high-throughput experiments on monkeys difficult to justify[5]. Rodents, with their increasingly well-understood cognitive and learning capabilities, have emerged as an alternative model system for studying a variety of complex behaviors[6–15]. Rats and mice share the basic mammalian brain architecture with primates, and though cortical specializations may differ[16–18], recent studies suggest that many of the well-characterized cortical functions in primates have equivalents in rodents[7,9,12,13,15,19]. Sophisticated tools for measuring and manipulating brain activity[20–23] together with the many transgenic lines and disease models available in rodents further incentivize their use in mechanistic studies. Yet one of the main barriers for research on complex behaviors, both in rodents and primates, lies in training animals - a process typically done under close supervision of researchers who frequently modify protocols and procedures on an animal-by-animal basis to improve learning rates and performance. This approach is labor-intensive and time-consuming, and makes the interaction between animal and researcher an integral part of the training process, possibly confounding comparisons of experimental outcomes across animals and labs[24]. 

Here we describe a method and experimental infrastructure that fundamentally transforms this traditionally arduous process, making it effortless, rigorous and amenable to high-throughput approaches. Our solution combines two main ingredients.

1. *Automation of the training process*. Automation allows implementation of rigorously defined training protocols on a large scale without the vagaries and efforts associated with human-assisted training[25]. Such improvements in the quality and quantity of behavioral data enables powerful research approaches for addressing complex and slow-to-learn behaviors [19].
2. *Training within the animal’s home-cage*. Implementing automated training within the animal’s home-cage eliminates the need for day-to-day handling of trained animals, making long training processes fully automated and largely effortless. Live-in home-cage training also enables long-term tethering of animals, making long-term uninterrupted neural recordings feasible.

While certain aspects of animal training have been automated, either through the use of custom-developed software or commercially available systems[19,26–30], most solutions lack the flexibility required to tackle broader sets of questions or behaviors. Existing solutions do not accommodate either complete automation of multi-stage training processes involving large numbers of animals or long-term neural recordings in the context of training. Thus the significant human involvement currently required for experiments on complex behaviors still represents a considerable impediment to large-scale rodent studies. 

**Figure 1:**
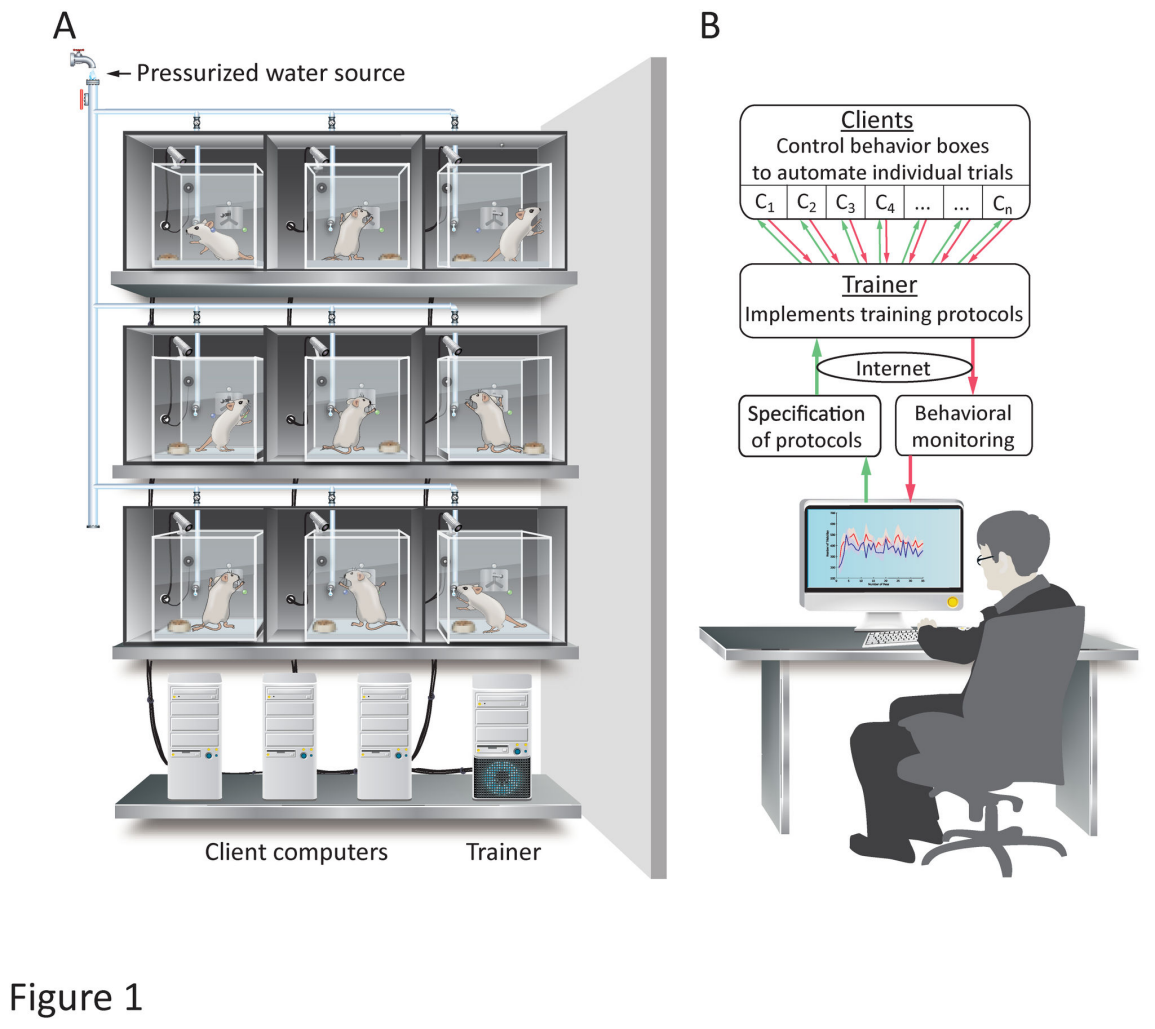
A fully automated high-throughput training system for rodents. **A**. Schematic of the hardware implementation. Custom-made behavior boxes, housing individual rats, are outfitted with task-specific sensors, manipulanda, and effectors. Water reward is delivered through a computer-controlled solenoid valve connected to the animal facility’s pressurized water supply. Client computers (2 boxes per computer) directly control and monitor the behavior boxes via a data acquisition card, using rules supplied by the Trainer. **B**. Logic of the software implementation. Training protocols and behavioral monitoring is implemented through a flexible and hierarchical software architecture. Clients (c) control individual trials by directly interfacing to a behavior box. The Trainer monitors overall performance and implements the training protocol by informing Clients about the rules and structure of individual trials. The user controls and monitors training remotely over the internet via a graphical user interface. Green arrows denote information flow regarding the training protocol; red arrows represent flow of behavior data.

To further improve the efficiency of the training process, we developed a fully Automated Rodent Training System (ARTS) for reward-based learning (Figure 1). Our low-cost system is flexible, extensible, remotely administrable, and allows for simultaneous training of large cohorts of animals. ARTS is designed for deployment in animal facilities found in biomedical research centers, and requires no more human supervision than standard animal care. Training occurs in custom-designed home-cages that can be outfitted with a variety of sensors, manipulanda, water ports, and effectors (e.g. sensory stimulation devices), customized to the nature of the behavioral task (Figure 1A; see Video S1 for a demonstration). 

Below, we outline the general architecture and logic of ARTS and its current implementation and describe, validate, and benchmark its use for motor learning in rats. 

## System Architecture

### Flexible and modular software architecture for control of high-throughput animal training

The heart of our automated rodent training system is the software platform that interacts with individual home-cages and executes pre-specified training protocols (Figure 1B, Figures S1- S3 in File S1). To allow for maximum flexibility and generality, the software suite is modular and hierarchical, with two different components controlling distinct aspects of the training process (Figure 1B). At the top of the hierarchy is the *Trainer*, which monitors overall performance and implements user-defined training protocols for individual behavioral boxes A ‘protocol’ is defined as a set of training stages and performance criteria for automatically transitioning between them (see Figures S5, S6, and Methods S1 in File S1 for details). Each training stage is specified in the form of a finite state machine (FSM), a widely employed and intuitive abstraction for specifying behavioral tasks that consists of states linked by transitions[31]. Behavioral or environmental events, such as lever presses, nose pokes, or elapsed time, can trigger transitions between states, each of which can be associated with a set of actions (e.g. reward being dispensed, LEDs turning on/off). 

The *Trainer* executes training protocols by supplying FSMs specifying reward contingencies and trial structure to lower level *Clients*, each of which controls a behavioral box. With this flexible and general program structure, automating a training protocol in ARTS reduces to having the *Trainer* supply the *Client* with the right FSMs at the right times. 

Data acquired by the *Client* during the execution of an FSM, including high-resolution timing data and video, is stored in a central database. This allows multiple users to concurrently and efficiently read and write data to and from the database using SQL - an intuitive database language supported by all major programming languages. Centralized data storage also allows for easy backup, aggregation, analysis, and distribution of large amounts of behavioral data. A software package for ARTS complete with a user’s manual can be downloaded from our server.

### Software Implementation

The entire software suite for ARTS is written in C#.Net, a simple, general-purpose, object-oriented programming language. The software (both source code and pre-compiled binaries) along with detailed step-by-step instructions on setting up the system can be downloaded from http://olveczkylab.fas.harvard.edu/ARTS (the system is co-branded OpCon in the website and internally). Both the *Client* and *Trainer* can easily be extended to accommodate virtually any behavioral task or training protocol by simply writing add-on custom-scripts in any .NET compatible language, including C#, F#, J#, VB, and C++. Writing or using these plugins does not require a detailed understanding of the underlying software (the plugins and scripts included in the source code can serve as a starting point). Importantly, the software supports numerous extensions to basic FSMs, like custom plugins and concurrent execution of multiple FSMs[31], which enables specification of behavioral paradigms with probabilistic cues and complex reward contingencies. 

In addition to the core components of ARTS (*Client*, *Trainer*, and the database), a suite of supporting software adds further functionality, making it a complete end-to-end high-throughput automated training system (Figures S1-S3 in File S1). A graphical user interface allows for easy and intuitive specification of training protocols. Behavioral monitoring, including querying the timing, duration, and saved video of each behavioral ‘event’ is made possible by an interactive data visualization tool. Furthermore, a suite of network services & scripts enables remote control and monitoring (including live streaming video onto the internet) of the system. 

### A cost-effective hardware solution

The hardware requirements of ARTS are modest, making it cost-effective and easy to build and deploy (Figure 1, Figure S4 in File S1). Our current system contains 48 behavior boxes controlled by 24 *Client* computers and two servers, all of which are housed in a temperature and humidity controlled animal facility. The reward port providing water reinforcement in each behavior box is connected to the animal facility’s pressurized water system through solenoid valves, allowing us to dispense specific volumes of water by controlling the duration of valve openings through the *Client*. Aquarium pumps (Jehmco LPH 60) are used to ventilate behavior boxes at a ratio of one pump for every six boxes. To ensure acoustic and visual separation each box is placed in an enclosure (Figure S4A in File S1), which is placed on the shelves of a standard wire racks.

Behavior boxes were custom designed using acrylic and aluminum extrusions. The boxes have a removable front panel holding all experimental equipment (sensors, indicators, manipulanda, water dispensing valves etc.; Figure S4A in File S1). The front panel is the only part of the system that needs to be customized for a given experimental paradigm. To ensure compatibility with invasive experiments (chronic electrophysiology etc.), the normal lid of the box can be exchanged with a custom lid having the experiment-specific equipment (e.g. commutator etc.). The hardware cost for building the boxes is ~$500/box. 

Each low-cost *Client* computer runs Windows 7 and contains an Intel quad-core processor (Core i5-750 - 2.66GHz/core), 4GB of DDR3 SDRAM, and a 1.5TB 5900 RPM hard disk and controls and communicates with the behavior boxes at the ratio of two boxes per computer. Behavioral data (e.g. from manipulanda, lick sensors, and cameras) and signals for controlling peripherals such as speakers and LEDs are transferred between the box and the Client computer via a National Instruments data acquisition system (NI PCIe 6323 - DAQ card, 2 x RC68-68 – Ribbon Cable & 2 x CB-68LP – Connector Block). The *Client* computers, in turn, communicate with a central server, which runs the *Trainer* and hosts the database (Figure 1). Server computers (*Trainer*) contain a higher-end Intel quad-core processor (Core i7-950 – 3.06GHz/core), 12GB of DDR3 SDRAM, 3 1TB 7200 RPM hard disks. The servers run Windows Server 2008 R2 and host a SQL Server 2008 R2 database.

The total cost of our current ARTS set-up (48 boxes), including all hardware, computers, and electronics, is around $67K, i.e. $1400/box (see Table S1 in File S1 for a breakdown of the costs and the “Hardware” section of http://olveczkylab.fas.harvard.edu/ARTS for a detailed list of the components of ARTS). Video S1 shows ARTS deployed in our animal facility. Detailed designs and specifications of the hardware implementation are available upon request. 

### Scalability and Safety

The number of behavior boxes per *Client* computer is primarily limited by the number of video cameras attached to each box since acquisition/processing of video data is fairly CPU intensive. ARTS supports multiple data acquisition cards per *Client* computer allowing a large number of behavior boxes to be controlled via a single computer if the *Client* does not need to process video data. Likewise the network bandwidth is dominated by uncompressed video data (17MBps per 30fps 640x480 webcam). The server can be scaled to support a larger number of clients by increasing the amount of available memory since this is the bottleneck for database performance.

In addition to free water at the end of every night (see section *Schedules and mode of reinforcement*) multiple layers of security checks are built into the system to prevent animals from dehydrating. Water is only dispensed upon licking the reward spout ensuring that the dispensed water is consumed. The behavior monitoring GUI prominently displays water consumption. A watchdog program continually monitors the *Client*, *Trainer* and database to detect any failures and displays this information in the monitoring GUI. Finally animals are periodically examined and weighed by the animal care staff to ensure they are adequately hydrated.

## Behavioral Training Methods

**Figure 2:**
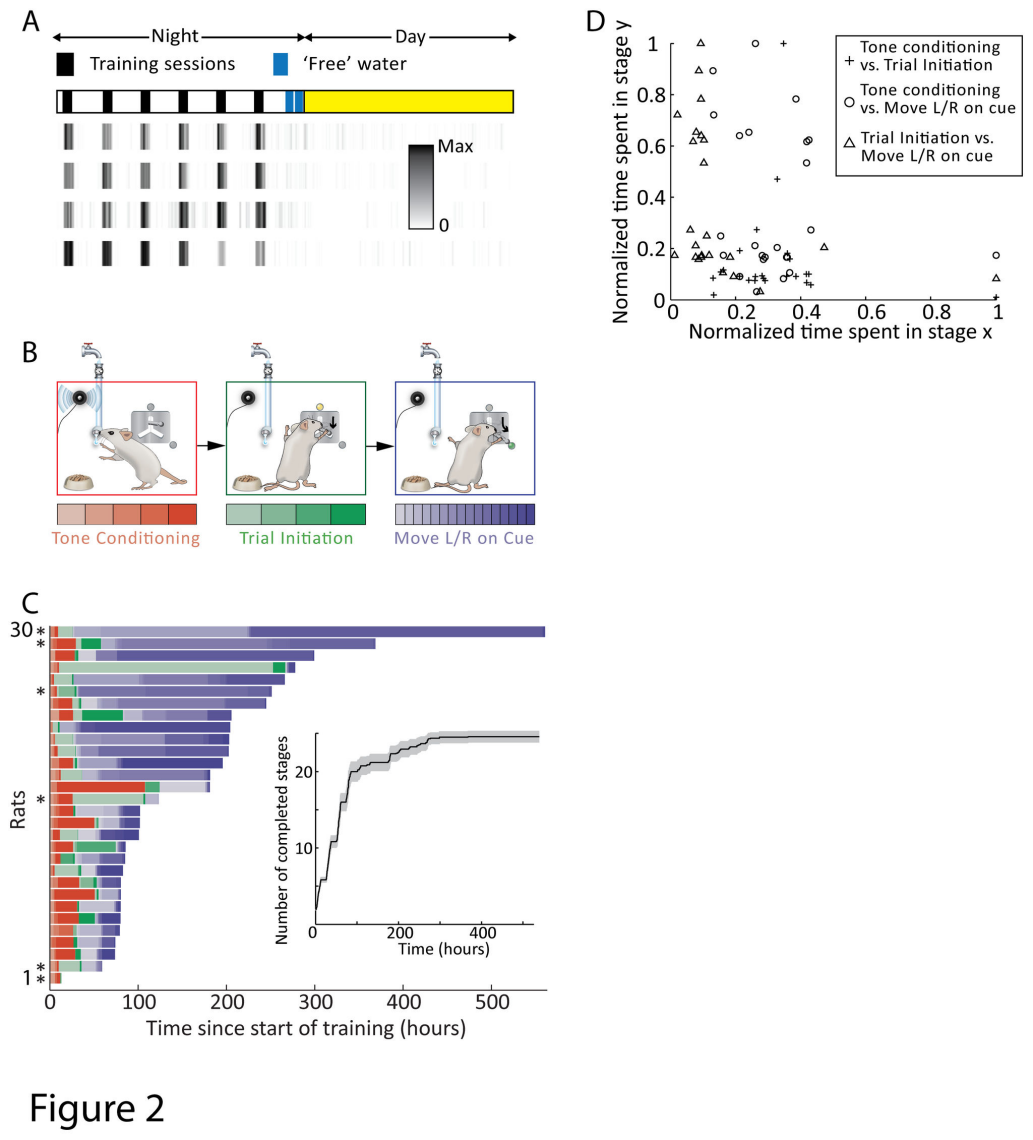
Complete automation of a complex multi-step training protocol for a version of the center-out movement task (see Methods). **A**. Training session structure. Animals were trained during six nightly 30-minute training sessions. The density plots show the distribution of joystick presses for four representative animals in their third week of training. ‘Free’ water is only available to rats earning less than a minimal amount of water during the nightly training session. **B**. Thirty rats were trained to perform the center-out movement task in three successive stages, each with multiple sub-stages (see Methods S1 in File S1 for details). Stage 1: touching the joystick for a reward tone and subsequently licking at the water spout to initiate water reward delivery. Stage 2: moving the joystick down on cue. Stage 3: moving the joystick left and right. **C**. Stage and sub-stage completion times for each rat. Six rats (indicated with asterisks) were dropped from the study due to poor learning. Inset shows the mean (and standard error) of the number of completed sub-stages as a function of time. **D**. Time needed to complete one stage vs. another for the 24 successful rats.

**Figure 3:**
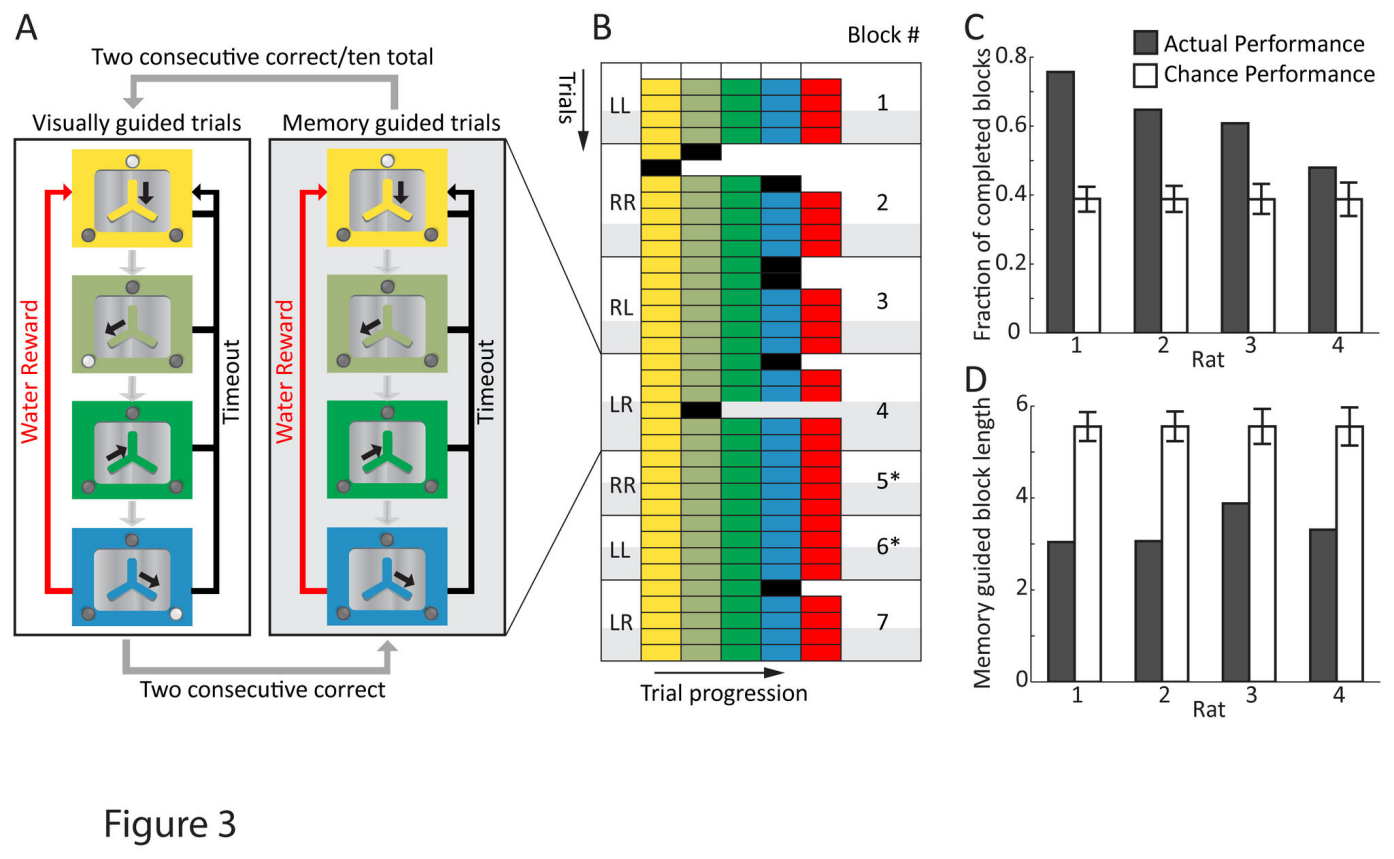
Automated training of memory guided action sequences. **A**. Structure of the behavioral task. An experimental block starts with a visually guided trial (left), in which the center LED indicates trial initiation. Upon moving the joystick down, the left (right) LED comes on. After a successful left (right) movement the LED turns off and the joystick is moved back to the center. A second movement is then cued by the right (left) LED. Upon moving the joystick right (left) a water reward is delivered. Any erroneous movement results in a timeout. After two consecutive correct trials, directional cues are not given and the movement sequence has to be performed from memory (right). After two consecutive correct memory guided trials (or ten total trials – an incomplete block), the next block commences with a new sequence. **B**. A sample run of 7 consecutive blocks from one animal. Each row represents one trial, with the sequence of movements color coded as in ‘A’. Left column denotes the target sequence (L-left movement; R-right movement). Shaded trials denote memory guided trials. Blocks denoted with asterisk correspond to perfect performance. Video S2 shows experimental blocks 5-7. **C**-**D**. Aggregate performance of 4 rats trained in the task as measured by the fraction of completed blocks (**C**) and the number of memory guided trials until completion (**D**). Performance is compared to simulated chance (error-bars denote 95% confidence level). Data from 679 blocks for Rat 1, 647 blocks for Rat 2, 472 blocks for Rat 3 and 392 blocks for Rat 4.

**Figure 4:**
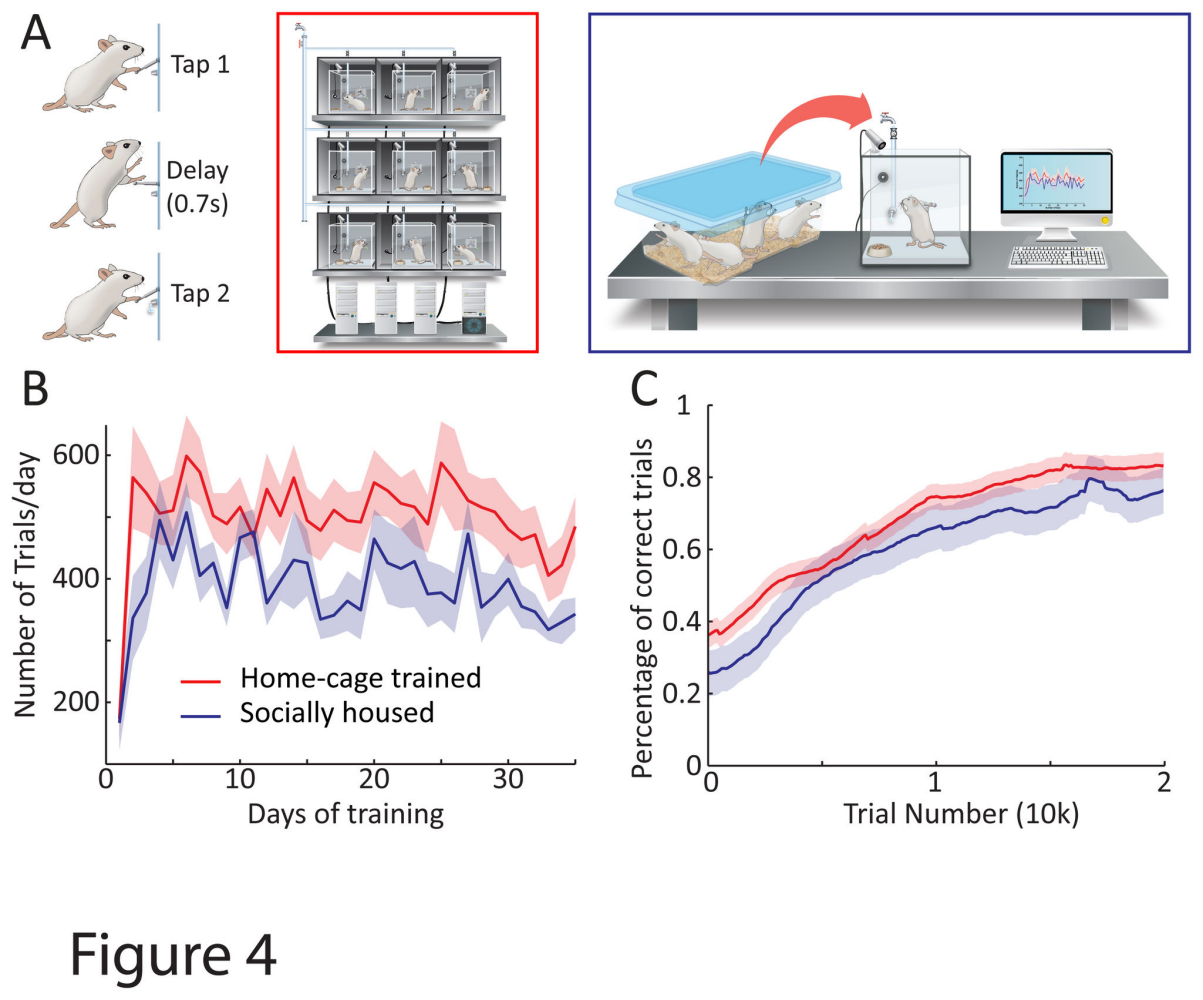
Fully automated live-in home-cage training is comparable to existing methods in terms of learning and performance. **A**. Rats were trained to spontaneously press a lever twice with a 700 ms delay between presses in an individually housed live-in training paradigm (red, n = 24 rats) or in a socially housed setting in which they were transferred to the behavior apparatus for daily training sessions (blue, n = 13 rats). **B**. Motivation as measured by the number of trials per day over time. **C**. Learning performance as measured by the fraction of correct trials, defined as trials within 30% of the 700 ms target inter-press interval, over time. Shaded regions in B and C represent standard error across animals.

We have used *ARTS* to train more than 150 rats in a variety of behaviors including pressing a lever in precise temporal sequences, pressing a set of levers in spatiotemporal sequences on cue, and moving a joystick in various directions on cue. In this report we focused on a subset of 67 female Long Evans rats aged ~10-12 weeks at the start of the experiment, 30 of which were trained to perform a simplified version of the center-out task (Figures 2,3) and 39 on a precise lever pressing task (Figure 4). Animals were kept on a daily 12h light cycle. 

### Ethics Statement

All experimental procedures were approved by the Harvard University Institutional Animal Care and Use Committee (protocol number: 29-15).

### Schedules and mode of reinforcement

Naïve rats were water-deprived for 8-10 hours before being transferred to their behavior boxes. After this, rats were trained for the next several weeks automatically by *ARTS* with no human involvement in the day-to-day training process. For the tasks in Figures 2 and 3, rats had 30-minute training sessions during their subjective night every 2 hours for a total of six training sessions. For the task in Figure 4, rats had 2 60-minute sessions per night. At the end of the night, ARTS automatically dispensed free water up to their daily minimum (5ml per 100g body weight). Rats also had a rest day every week during which water was dispensed *ad libitum*. Blinking house lights, a continuous 10s 1kHz pure tone and a few drops of water marked the beginning of each training and free water session.

### Center-out task

Rats were trained to move a 2D joystick left/right along two arms of an inverted-Y shaped slit (Figure 2). The equilibrium position of the joystick is at the top of the inverted-Y. Trial availability was indicated by the center LED; a rat could commence a trial by moving the joystick down by ~2 cm to the point where the two arms of the inverted-Y meet. Then, the left(right) LED turned on, and if the rat guided the joystick >5 cm along the left(right) arm of the slit, the trial was considered successful and a reward tone (1000Hz for 100ms) presented. The rat could then lick the reward spout to collect water and commence the next trial. If the rat moved in the wrong direction, a 7 second timeout was instituted before the next trial could be initiated.

### Precise lever pressing task

Rats were trained to press a lever twice with a 700 ms delay between the presses. Animals could self-initiate the trial by pressing the lever. After learning to associate lever pressing with water, rats were rewarded for increasingly precise approximations to the target sequence, i.e. 2 lever presses separated by 700 ms. The reward contingencies were automatically updated based on performance to ensure that, on average, ~30-40% of the trials were rewarded. If the trial was successful, a reward tone was played and water reward dispensed upon licking of the reward spout. Animals had to wait 1.2s before initiating the next trial if the inter-press interval fell outside the rewarded range.

## System Validation

ARTS is designed for fully automated training of complex behaviors in a home-cage environment and thus represents a significant departure from current practice. The day-to-day interaction of the researcher with experimental animals and training apparatus is eliminated as is the need for transferring animals back-and-forth between procedural chambers and holding rooms. Whether completely replacing the researcher with contextual cues can successfully get animals to learn complex tasks has not been evaluated. To ensure the feasibility of our approach as a general purpose solution for large-scale rodent training, we have done extensive testing and characterization of the system, including training more than hundred and fifty animals in a variety of sensorimotor tasks, a subset of which we report on below. 

### Structure of fully automated training

A major challenge presented by home-cage training is motivating animals to perform the behavioral task. In traditional reward-based training paradigms[10,15,27], the experimenter places a water- or food-deprived animal in the behavioral apparatus and rewards correct behaviors with liquids or foods. As hunger or thirst is satisfied, the researcher removes the animal and commences deprivation anew. We automate this process within the animal’s home-cage by dispensing water as reward only during training sessions, the start and end of which are indicated to the animal by a set of salient sensory cues (e.g. flashing house lights). Time between sessions serves to deprive the animal of water and thus build up motivation for the next session. We have deployed a variety of session structures. For the center-out movement task described below (Figure 2B), for example, animals had 6 30-minute training sessions per day spaced at 2 hour intervals (Figure 2A), whereas for the task in Figure 4, we employed 3 daily 60-minute sessions, each separated by 4 hours. Whether a particular session structure is superior to others has yet to be rigorously tested, but our experience thus far suggests that this is not a critical parameter.

Animals quickly learn to engage with the task (e.g. manipulating a joystick) predominantly during specified training sessions: in the third week of training in the center-out task, the likelihood of a rat pressing the joystick was, on average, 24 times higher in-session than out-of-session (n = 24 rats; Figure 2A). To prevent poorly performing animals from dehydrating, water is provided at the end of the night for animals that do not receive the prescribed minimum daily water amount during training.

### Validation of ARTS: Center-out movement task

A standard paradigm for studying neural control of movement in primates is the center-out reaching task[32,33], which involves moving a manipulandum to one of several possible cued locations. Rats trained with traditional methods can master a version of this task in a matter of weeks[15]. In our implementation of the task, rats are required to move a two-dimensional joystick along the arms of an inverted Y-shaped slot with their forepaws (Figure 2B, Methods). Trials are initiated by moving the joystick down the vertical arm of the slot in response to an LED cue. A second LED then prompts the animal to move the joystick either left or right. A correct trial is indicated by a short tone followed by water reward. Thirty rats were trained on the task in three sequential stages, each containing multiple sub-stages (Figure S5 and Methods S1 in File S1). All but one rat completed the first training stage (touching the joystick for a reward tone and collecting water reward) within 12 hours. Twenty-four out of 30 rats completed the second (moving the joystick vertically down on cue) and third (moving the joystick left and right) training stage to criterion (Figure 2C, Methods). Despite being trained using the same training protocol, animals learned at different rates (time to complete all three stages = 148 ± 78 hrs (mean ± S.D.) from start of training, range = 73 - 299 hrs, n = 24 rats). Furthermore, learning rates on one training stage was not a good predictor for mastery of other stages, which involved different sets of cognitive, learning and motor control challenges. The correlation coefficients between the time to complete different stages were -0.05 (stage 1, 2), -0.18 (stage 2, 3), and -0.34 (stage 1, 3) respectively (n = 24 rats; Figure 2D). Faced with such a substantial variation in the speed of learning across subjects and in distinct phases of learning, studies on complex learning that use learning rate as a behavioral readout will require large cohorts of animals trained in identical tasks, an approach that will be much helped by automated high-throughput training systems. 

### Validation of ARTS: Memory guided motor sequence execution

Having the capacity to simultaneously and effortlessly train large groups of animals, reduces the risk associated with - and the investment made in – individual animals, making it feasible to train even very challenging tasks, i.e. ones that only a small fraction of animals may be capable of learning. We deployed our automated training set-up to explore whether rats can master sophisticated motor sequence learning paradigms previously used only in primates[2]. In particular we were interested in the extent to which rodents can execute action sequences from working memory[34]. We trained the 4 best rats in the center-out task (Figure 2) to make sequences of left/right joystick movements from memory (Figure 3A, Figure S6 in File S1). At the beginning of each block of trials, visual cues (LEDs) were used to instruct the correct sequence of movements. After 2 consecutive correct visually guided trials, cues were removed and animals had to perform the same movement sequence from memory. Rats progressed to the next block (i.e. new sequence) after 2 consecutive correct memory guided trials or 10 trials, whichever occurred first.

Figure 3B shows an example of the star performer in this task once asymptotic performance was reached (see also Video S2). The errors in the visually guided trials at the start of some blocks are typically due to the animal performing the sequence from the prior block. 

To measure the extent of learning, we compared a week’s performance on the task to simulated chance (Figures 3C,D), modeled as random left/right movements during the memory guided trials. All 4 rats completed significantly more blocks (i.e. got 2 consecutive correct memory guided trials within a span of 10 trials) than expected by chance (Figure 3C, fraction of completed blocks = 63% vs. 39% by chance; probability of observing performance by chance < 2e-4). Furthermore, the average number of memory guided trials required to complete a block (which can range from 2 to 10) was substantially smaller than chance levels for each animal (Figure 3D, 3.2 vs. 5.4 for chance; probability of observing performance by chance < 1e-4). The best rat completed over 75% of the blocks with, on average, only 3 memory guided trials per block. These results validate rodents as a model for working memory guided motor sequence generation, and ARTS as an efficient method for training such complex behaviors. 

### Benchmarking home-cage training against existing training methods

To benchmark the live-in training concept against more traditional methods, we compared the performances of rats trained in our home-cage set-up (n = 24 rats) with ones housed in social groups and exposed to the behavior apparatus only during daily training sessions (n = 13 rats) (Figure 4A). Both groups were trained in identical behavioral boxes using the same automated training protocol. Rats were trained to spontaneously press a lever twice with a 700 ms delay between presses (Figure 4A, Methods). Motivation to do the task, as measured by the number of trials initiated per day, was similar between the two groups (494 ± 243 (mean ± S.D.) trials per day for automated training vs. 426 ± 123 trials per day for manual training on day 15 of training; Figure 4B). Furthermore, learning rates, as characterized by the fraction of ‘correct’ trials (defined here as inter-press intervals within 30% of the 700 ms target) at 20,000 trials was also comparable (83% ± 8% for automated training vs. 76% ± 17% for manual training, p=0.22; Figure 4C). Beyond demonstrating the feasibility and non-inferiority of live-in training in terms of performance, our results also validate the use of our automated training system in cases when rats are transferred to behavior boxes only for the duration of training. While home-cage training has the obvious advantage of requiring no human involvement other than standard animal care, there may be scenarios in which the benefits of fully automated home-cage training outweigh the negative effects of social isolation[35,36]. In such instances ARTS can still automate all other aspects of training (as was done for the socially housed cohort in the precise lever pressing task, Figure 4). An added benefit of manually transferring animals to the behavior box during training sessions is that the same box can be multiplexed across many animals increasing the throughput of the system[19,26].

## Discussion

We present a cost-effective, modular, and fully automated training system for rodents (ARTS, Figure 1) that dramatically decreases the effort required for implementing operant learning paradigms. Deploying the system in our animal facility enabled high-throughput training of rats with performance and learning rates similar to more traditional methods (Figure 4). While we benchmarked our system in a variety of motor learning tasks (Figures 2, 3 and 4), we believe that its flexibility, modularity, and extensibility ensures that it can be used to automate virtually any training protocol relying on reward-based learning. 

Though we designed and benchmarked ARTS for rats, a simple modification to the geometry of the home-cage should make the system applicable also to mice, though the extent to which mice are amenable to fully automated training in our system remains to be seen.

Simple behavioral tests in rodents have revolutionized our understanding of neurological function by allowing large-scale phenotyping of experimental animals[37]. Automated training further extends the power of rodent models in neuroscience by enabling standardized high-throughput studies of more complex behaviors[30]. Full automation also removes the vagaries inherent to human-assisted training by requiring explicit codification of all training steps, including contingencies and criteria for progressing from one stage to the next (example in Figures S5, S6 and Methods S1 in File S1). Such a compact and complete description of the training process makes reproducing and comparing experimental outcomes across different animal cohorts and labs possible and meaningful. 

Automated training protocols not only standardize the training process, but they ensure that incremental insights and improvements to training strategy accumulate. Indeed, our experience in setting up novel training tasks is that their implementation improves with time, as inefficiencies and ‘bugs’ in the training protocol get sorted out. In contrast to human-assisted training, where these experiential gains are largely confined to the researcher, automated training ensures that each improvement becomes part of an ever-evolving protocol.

Having well defined discrete training stages, each associated with its distinct set of cognitive, learning, perceptual, or motor control challenges, also enables increased specificity of the behavioral analysis. For example, when we analyzed learning rates in different phases of a multi-stage task we found no correlation between them, meaning that facility with associative learning aspects of a task, for example, may not translate into success on motor learning aspects (Figure 2D). Breaking down the learning process to its elementary components by evaluating each training stage independently will permit a more detailed phenotypic analysis and thus help better pinpoint how specific manipulations, genetic or otherwise, impact learning and performance of complex multi-faceted behaviors.

The advantages of home-cage training go beyond the benefits of full automation. It eliminates animal handling and the performance variability that goes with it[25] and fully automated continuous long-term neural recordings in behaving animals a feasible prospect. An initial practical concern with home-cage training, however, was the possible impact of social isolation on learning and performance[35,36]. In our benchmarking, however, we did not see a difference in either motivation or learning rates as compared to animals that were housed socially and exposed to the behavioral chamber only during training (Figure 4). It is possible that any detrimental effect of social isolation is compensated for by other factors unique to automated training, such as precise and regimented training schedules. Further experiments are needed to fully characterize the effects of social isolation on motivation and learning in a home-cage setting, with the understanding that different tasks may be impacted differently. 

Lowering the barrier for training large number of animals on complex behavioral tasks, as ARTS does, has the potential to accelerate research towards understanding many fundamental questions in neuroscience.

## Supporting Information

Video S1 **A 2m30sec video highlighting the functionality and features of ARTS, and showing its deployment in our animal facility.**
(MP4)

Video S2 **Video of a rat performing the task shown in Figure 4.** The video contains the stretch of trials corresponding to Blocks 5-7 in Figure 4B. On the right of the movie file is seen the joystick trajectory. Colored cues shown in the video correspond to cues seen by the animal (obscured in the video). Red square corresponds to the cue for initiating a trial. Green square denotes the cue for pushing joystick to the right; blue square for pushing the joystick to the left.
(MP4)

File S1 Methods S1. Table S1. Figures S1-S6.
(PDF)
