# Supplementary material for: A Fully Automated High-Throughput Training System for Rodents
Source: PLoS One. 2013 Dec 6;8(12):e83171. doi: 10.1371/journal.pone.0083171 (PMC3857823; doi:10.1371/journal.pone.0083171)
Supplement: File S1 — Methods S1. Table S1. Figures S1-S6. (PDF) [file pone.0083171.s003.pdf]

Supplementary Methods

Training stages for the center-out task

Rats were trained in three sequential stages, each consisting of multiple sub-stages. Progression required performing a pre-specified number of correct trials at a stage. The first stage acquainted animals with the behavior box, which included the following sub-stages (numbers refer to Fig. 2):

- collecting water within 3s of it being dispensed from the reward spout (1_0);

- licking the reward spout causing water to be dispensed (1_1);

- collecting water within a specified time interval after a sound (1 kHz pure tone, 200 ms long) is played (sub-stages 1_2, 1_3, and 1_4 associated with decreasing frequency of tone playback and time-intervals)

- touching the joystick to produce the reward tone and subsequent reward delivery (1_5).

The goal of the second stage was to get rats to press the joystick down by 2.5cm when the center LED was lit. Rats were gradually shaped to do this by increasing the required amplitude of movement over five sub-stages (2_0 – 2_4). Finally the rats were trained to press the joystick down when the center LED was on (2_5).

The third stage shaped rats to move the joystick left or right along the arms of the inverted Y, after moving it down, to perform the final task. At the beginning of the third stage (3_0) both cues (left and right) appeared with equal probability, there was no timeout for moving in the wrong direction and rats needed to move the joystick only 0.5cm in either direction. The cue frequencies, required amplitudes of movement, and the timeouts associated with incorrect joystick movements were gradually changed in 16 small steps (3_0 to 3_{15}) until the rat was moving the joystick to its full extent in both directions.

Training Stages for the precise lever pressing task

Water deprived rats were initially trained to associate the water spout with a water reward by dispensing 'free' water. After this they were trained to associate a short 100ms pure tone with availability of a water reward which was dispensed upon licking the water spout. Next, they were required to press the lever to trigger the reward tone and then to press the lever twice in succession for the same reward tone. Finally they were trained to wait 700ms between consecutive lever presses by gradually narrowing the reward window around this target interval.

At the beginning of each training session, the rewarded inter-tap interval range was calculated based on the previous session's performance, such that animals received reward on ~ 35-40% of the trials.

Supplementary Videos

Video 1: A 2m30sec video highlighting the functionality and features of ARTS, and showing its deployment in our animal facility.

Video 2: Video of a rat performing the task shown in Fig 4. The video contains the stretch of trials corresponding to Blocks 5-7 in Fig. 4B. On the right of the movie file is seen the joystick trajectory. Colored cues shown in the video correspond to cues seen by the animal (obscured in the video). Red square corresponds to the cue for initiating a trial. Green square denotes the cue for pushing joystick to the right; blue square for pushing the joystick to the left.

Supplementary Table 1

**Component**	**Unit Cost**	**Qty**	**Total Cost**
Client Computers (Core i5-750, 4GB RAM)	$700	24	$16,800
NI - DAQ System - (NI-PCIe 6323, 2x RC68-68, 2x CB-68LP)	$800	24	$19,200
Server Computers (Core i7-950, 12GB RAM)	$2,000	2	$4,000
Custom behavior boxes (Plastic, LEDs, valves, etc.)	$500	48	$24,000
Common Infrastructure (Wire shelving, water supply system, audio-visual isolation boxes, etc.)	$3,000	1	$3,000
**Total**	**$1,395.83**	**48**	**$67,000**

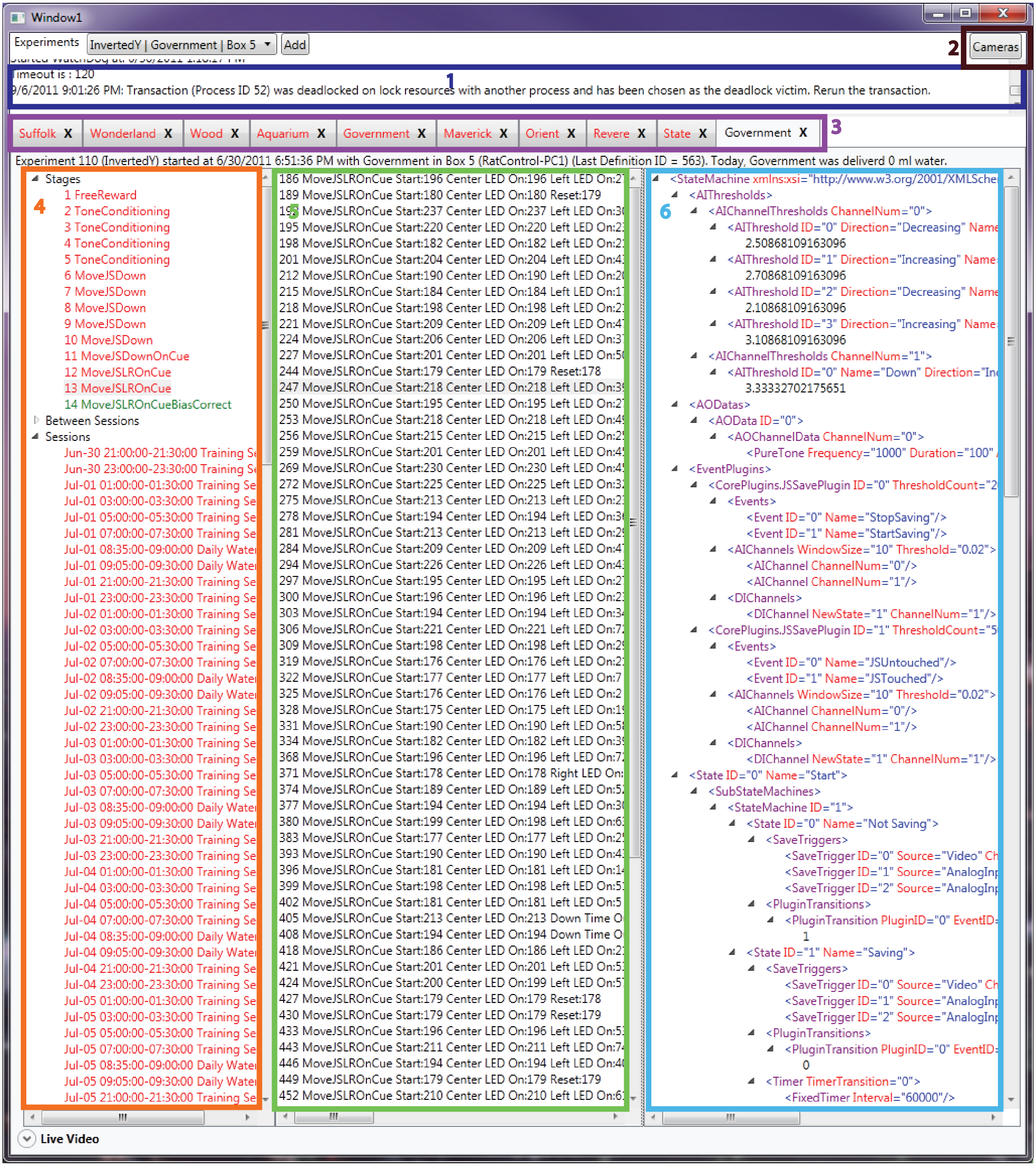

Supplementary Fig 1 A screenshot of *ARTSMonitor*, the behavior monitoring GUI of *ARTS*. This window shows an overview of behavior data for each rat being trained. 1. Messages from *ARTSWatchDog*, a program that continually monitors the entire system to make sure it is operating smoothly. 2. Allows the operator to view live streamed video of every rat. 3. Each tab in the window shows more detailed behavior data for each rat being trained. Critical pieces of information like how much water the animal received in the past 24 hours, what stage of training it is currently at are immediately visible in this interface. 4. Groups finite state machines by training stage or training session. Selecting an entry in this panel shows all the finite state machines that were executed within that stage or session. 5. Shows the number of entries to each state of a finite state machine, giving an immediate picture of the number and fraction of various trial outcomes over time. Double clicking a finite state machine in this panel opens a new window that shows detailed trial by trial behavior data as shown in Supplementary Fig 2. 6. Shows the XML document specifying the detailed structure of the finite state machine selected in the middle panel.

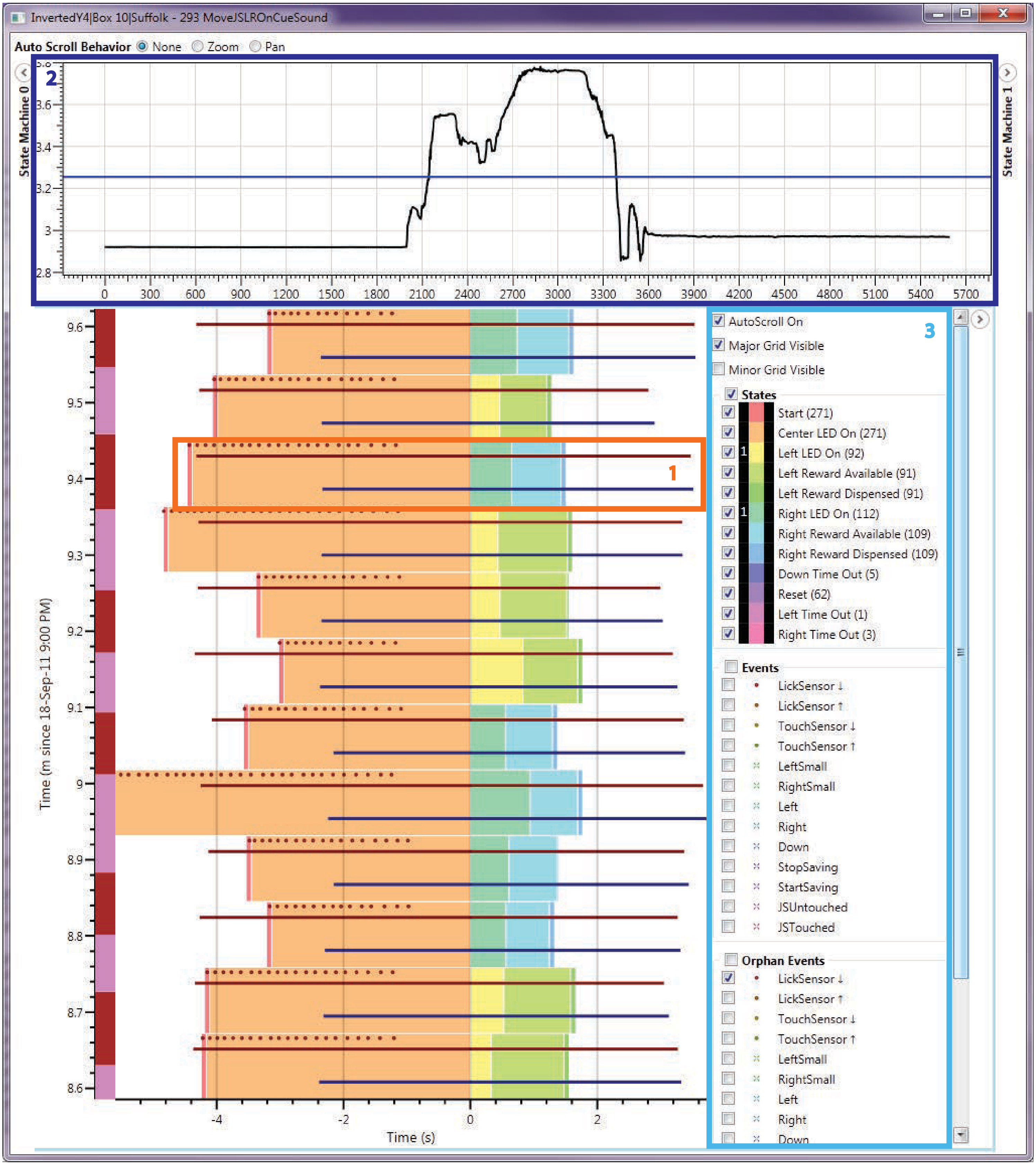

Supplementary Fig 2 A screenshot of the interactive data-visualization tool of *ARTS*. This window shows data from one finite state machine which can consist of several hundred trials over a 30-minute session. 1. Each row of the plot is a trial which comprises of a sequence of states each of which is identified by a color. Colored markers show the times of events (brown dots represent licking in this case) and colored lines show intervals for which some data was saved. Clicking on the brown lines allows viewing saved video from each trial. 2. Clicking on the blue lines shows the joystick trajectories on the top panel of the window. 3. This panel allows customization of the display by choosing the colors, marker styles and visibility of the various, state, intervals and events associated with the FSM being displayed. The display is highly interactive allowing interactive zooming, scrolling and alignment to onsets of chosen state entry events.

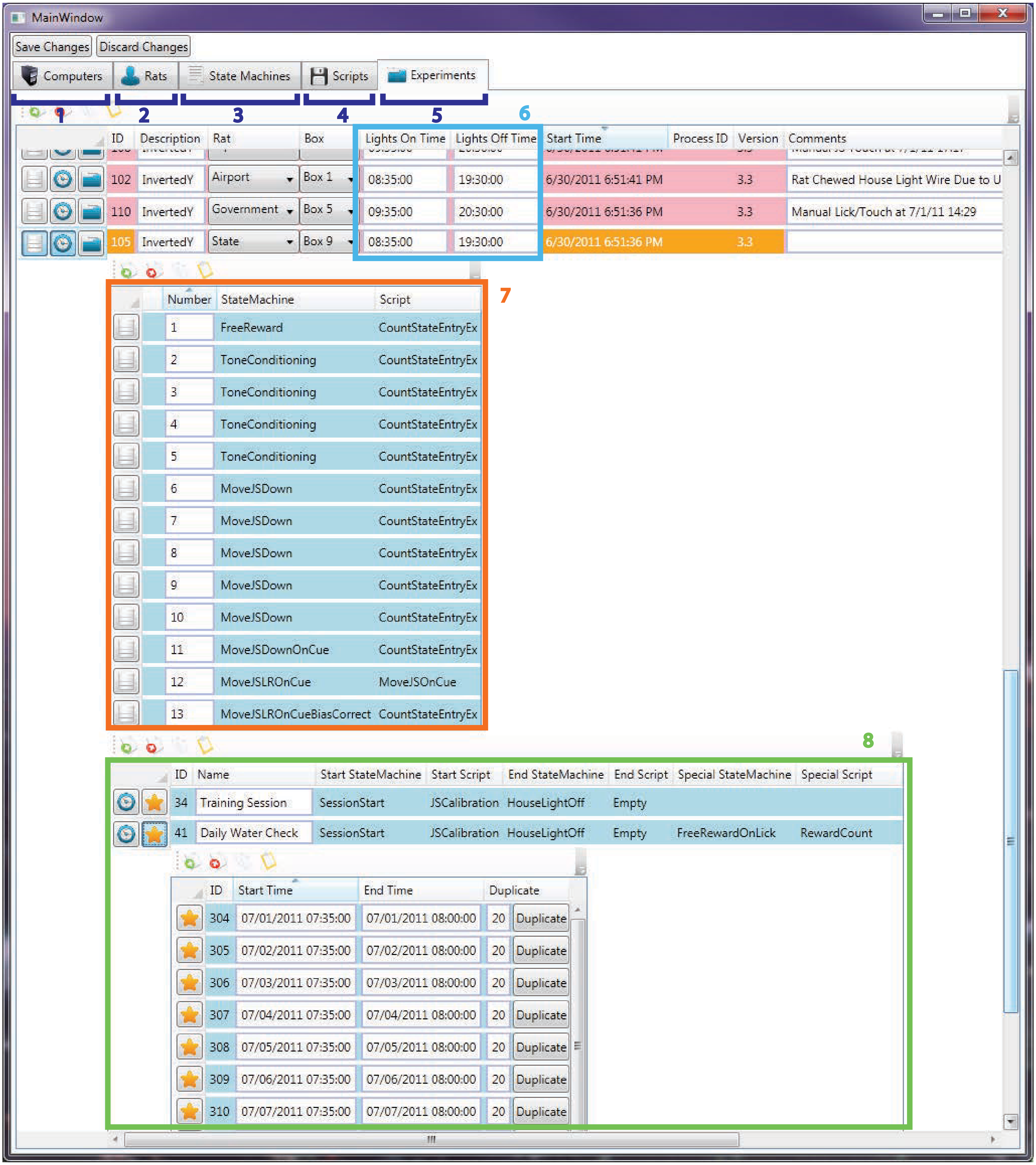

Supplementary Fig 3 A screenshot of *ARTSSpec*, the system configuration and protocol specification tool of *ARTS,* which provides a AGUI for interacting with the central *ARTS*database. The 'Computers' tab (1) allows configuring the various client computers comprising the *ARTS* system, and the behavior boxes connected to each computer. An XML document, the 'Box Definition', is associated with each behavior box and specifies the configuration of each box, like which actuators and sensors are part of the behavior box and which channels in the data acquisition card they connect to, making the system flexible enough to accommodate any behavioral paradigm. The 'Rats' tab (2) allows entering the names, weight, etc. of the animals being trained. The 'State Machines' tab (3) allows specifying the FSMs that correspond to each task automated by *ARTS* during training in the form of an XML document, which is a human readable and intuitive format for specifying Finite State Machines. *ARTS* contains XML schemas that specify the syntax of these documents allowing auto-completion and live syntax checking ('IntelliSense'), making entry of state machines user friendly. The 'Scripts' tab (4) allows specifying custom-written scripts that *ARTSTrainer* uses to evaluate behavioral performance and transition to subsequent stages if necessary. Finally the 'Experiments' tab (5), allows putting all this together, to specify the entire weeks-long training protocol which consists of the day/night light cycle (6), the FSMs and scripts associated with training stages (7), and the session structure (8).

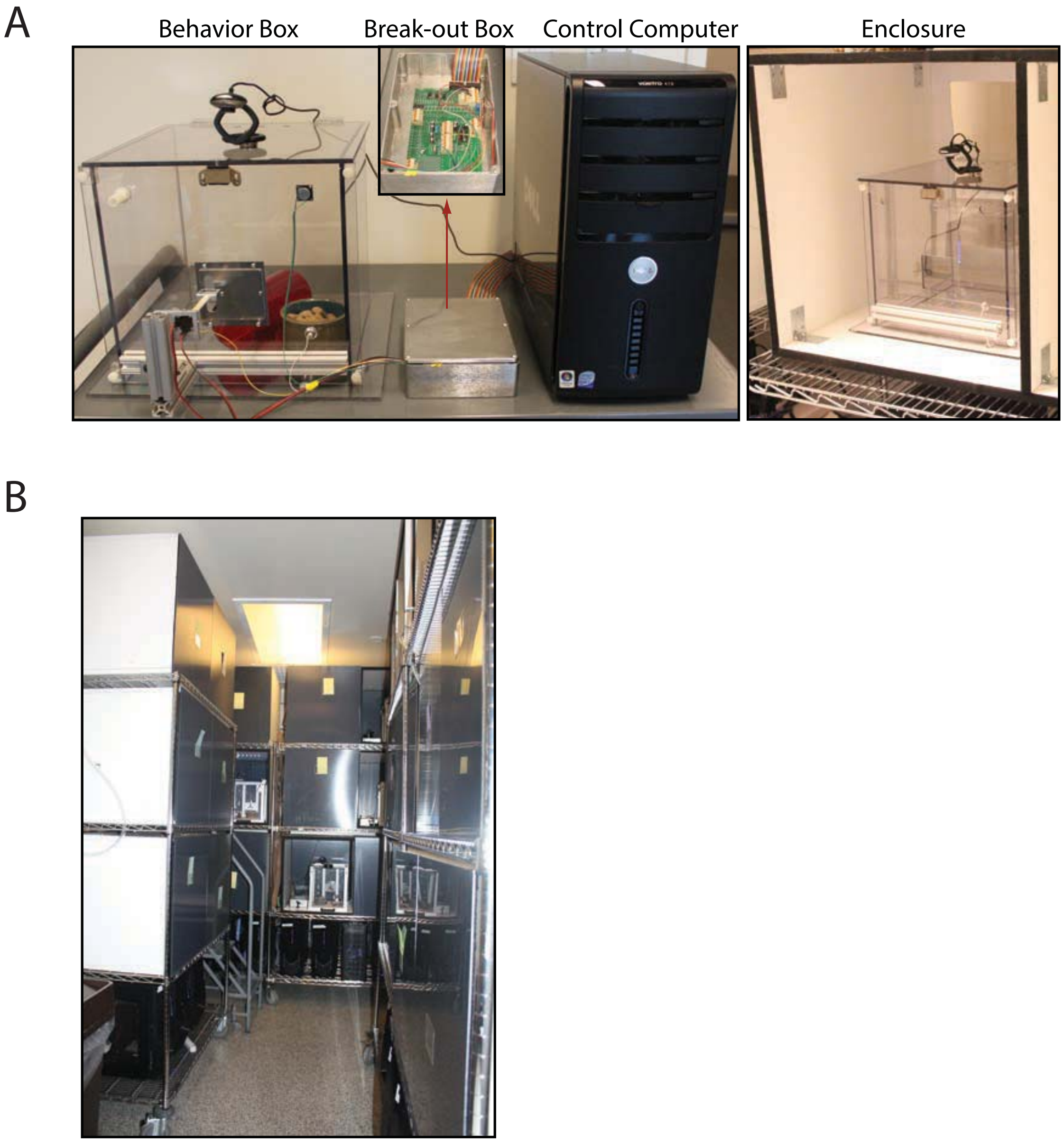

Supplementary Fig 4 Hardware implementation of our fully automated system. **A**. (Left) The behavior box is connected to the data acquisition card of the client computer via a general purpose breakout-box (see inset). (Right) The Box is housed in a custom-made isolation enclosure in the animal facility. **B.** Deploymenty of the system in our animal facility.

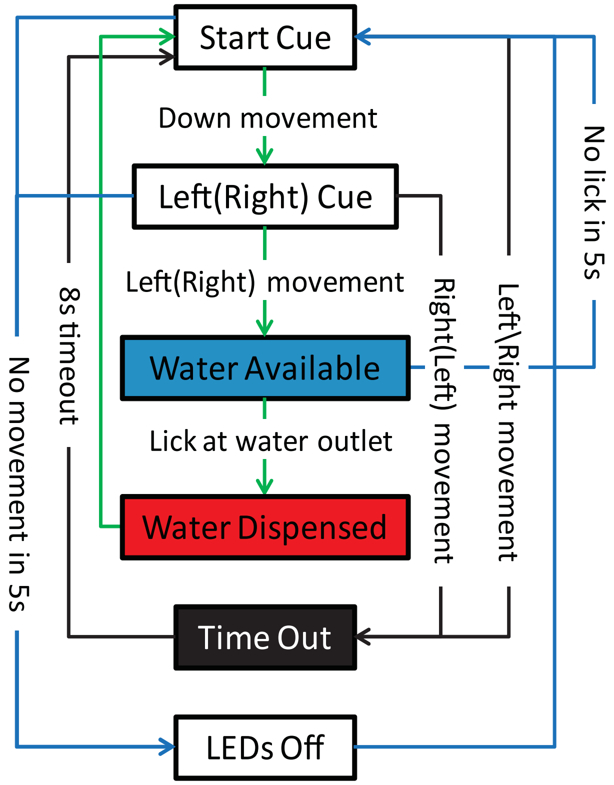

Supplementary Fig 5 Finite state machine for the center-out task of Fig 2. Each rectangle is a state and arrows indicate tranistion between states. The arrow labels specify the event causing the tranistion. Green paths correspond to a correct trials, red to incorrect and blue to abandoned ones.

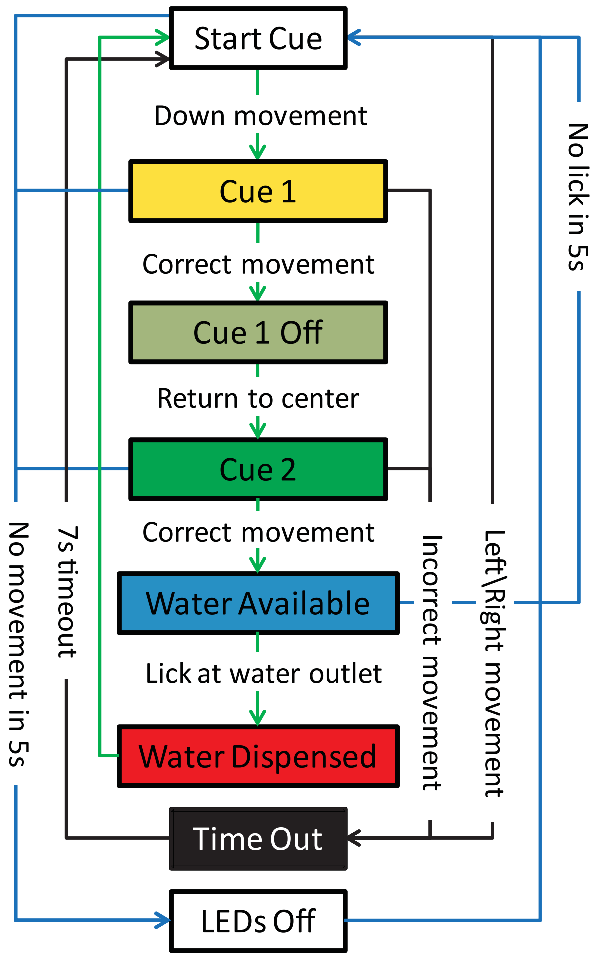

Supplementary Fig 6 Finite state machine for the movement sequence task of Fig 4. The color of the state rectangles matches the color scheme used in Fig. 4.
